# A rapid review of the causes of diagnostic and treatment delays for tuberculosis in low-burden countries

**DOI:** 10.1093/pubmed/fdaf106

**Published:** 2025-09-12

**Authors:** Louise Preston, Alexander Thompson, Susan Baxter, Duncan Chambers, Paul Collini, Louise Falzon, Jack Goodall, Andrew Lee

**Affiliations:** School of Medicine and Population Health, University of Sheffield, Western Bank, Sheffield S10 2TN, UK; School of Medicine and Population Health, University of Sheffield, Western Bank, Sheffield S10 2TN, UK; School of Medicine and Population Health, University of Sheffield, Western Bank, Sheffield S10 2TN, UK; School of Medicine and Population Health, University of Sheffield, Western Bank, Sheffield S10 2TN, UK; School of Medicine and Population Health, University of Sheffield, Western Bank, Sheffield S10 2TN, UK; School of Medicine and Population Health, University of Sheffield, Western Bank, Sheffield S10 2TN, UK; School of Medicine and Population Health, University of Sheffield, Western Bank, Sheffield S10 2TN, UK; School of Medicine and Population Health, University of Sheffield, Western Bank, Sheffield S10 2TN, UK; United Kingdom Health Security Agency, Quarry Hill, Leeds LS2 7UE, UK

**Keywords:** diagnosis

## Abstract

**Background:**

Delays in diagnosing and treating tuberculosis (TB) have significant implications. We undertook a rapid review to explore factors associated with delays at all stages of the diagnostic and treatment pathways in low-burden settings.

**Methods:**

We searched databases (Embase, Medline, CENTRAL, Cinahl, PubMed, Cochrane Database of Systematic Reviews, and Web of Science) for qualitative and quantitative evidence (2010–25) from countries with low TB burden (incidence rate <40/100 000 in 2020). Included studies were assessed on their robustness and relevance. Due to the rapid review design, we did not conduct formal quality appraisal.

**Results:**

The review included 3 reviews, 5 qualitative studies, 18 cohort studies, and 13 cross sectional studies (*n* = 41) with varying robustness and relevance. By synthesizing data using a patient pathway, we uncovered patient- and healthcare-related factors that contribute to delays such as medical history, health behaviours, level of patient and physician suspicion of TB, service location (primary care), and timing of TB testing. Having extrapulmonary TB was associated with greater total delay.

**Conclusions:**

We have identified patient and health service factors that are consistently associated with patient, diagnostic, and total delay from TB symptom onset to initiation of treatment in low-burden settings. Factors amenable to change should be the focus of public health interventions aimed at reducing TB diagnostic delay.

## Background

Tuberculosis (TB) is a leading cause of death globally. This is attributable to multiple factors, including the limited effectiveness of vaccination programmes, insufficient access to timely diagnosis and treatment, high mortality rates, and the persistent transmissibility of TB. Delays in diagnosing and initiating treatment for TB leads to worse outcomes for both patients and the health system. As a result, considerable attention has been directed towards understanding the factors associated with diagnostic and treatment delays.^1^ Although several reviews have examined these delays, the existing literature has predominantly focused on studies conducted in high-burden settings.^2^

Definitions of high- and low-burden TB settings can vary. The World Health Organization (WHO) defines low-burden settings as countries with a TB incidence < 10 per 100 000 people.^3^ In the UK, infants have historically been offered the Bacillus Calmette-Guérin vaccination if their parents were born in high-burden TB settings, defined as countries with an incidence rate exceeding 40 cases per 100 000 population.^4^ We maintained this threshold, with countries reporting incidence rates below this value being classified as low-burden settings.

The reduction in the global burden of TB requires action across low-burden countries as well as high-burden countries.^5^ While the UK has historically had a low-incidence of TB, recent increases in the incidence rate highlight the importance of avoiding complacency in TB management.^6^^,^^7^ The purpose of this work was to review evidence relevant to the UK to support commissioners and decision makers by addressing the following questions:

1. What factors are associated with delays in symptomatic patients seeking testing in low-burden settings?
2. What factors are associated with delays in testing, referral, and diagnosis of symptomatic patients with TB within health services in low-burden settings?
3. What factors are associated with delays in diagnosed patients receiving treatment for TB in low-burden settings?

## Methods

We carried out a mixed method rapid review of available evidence on factors associated with delays in diagnosis and treatment for TB in low-burden settings. This review was commissioned by the UK Health Security Agency (UKHSA).

### Search approach

Searches were undertaken in February 2023 using Ovid MEDLINE, PubMed, EMBASE, The Cochrane Library (Cochrane Database of Systematic Reviews and CENTRAL), CINAHL, and Web of Science. We also screened reference lists of included studies for additional evidence and carried out citation/similarity checking on key documents to seek other potentially relevant sources. We also screened the websites of relevant organizations including UKHSA, European Centre for Disease Prevention and Control, US Centers for Disease Control and Prevention, Robert Koch Institute, Public Health Agency-Canada, the WHO, and the International Union Against Tuberculosis and Lung Disease. Searches of Ovid Medline, EMBASE, The Cochrane Library, and CINAHL were updated in January 2025. Our search strategy is included as appendix five in the Supplementary material.

### Selection of included evidence

References were screened in the endnote at title level and abstract (where available) by one reviewer, with a second screening of the database split between two additional reviewers, ensuring full double screening. For references that were tagged for inclusion by one reviewer, full text articles were obtained and examined by all three reviewers with discussion to reach agreement regarding ultimate inclusion. Other team members with topic-specific expertise were consulted, when necessary, to establish consensus. Inclusion/exclusion criteria are presented in Table 1. Since the search included review articles, three of the studies we included also appeared in the content of those reviews.

**Table 1:** Inclusion and exclusion criteria.

Inclusion	Exclusion
Evidence published since 2010	Evidence published prior to 2010
Countries with low TB incidence (<40 per 100 000 in 2020)	Countries with high TB incidence (≥40 per 100 000 in 2020)
High income countries	Low income countries
Literature published in academic journal and reports published by relevant organizations	Literature in professional magazines, PhD theses
Evidence containing empirical data	Narrative or opinion-based evidence
Experimental, observational and qualitative designs including controlled and uncontrolled trials, reviews, prospective and retrospective cohort studies, economic analysis, epidemiological studies, qualitative studies	Individual case reports Conference abstracts
Pulmonary and extrapulmonary TB	

### Data extraction and synthesis

**Figure 1:**
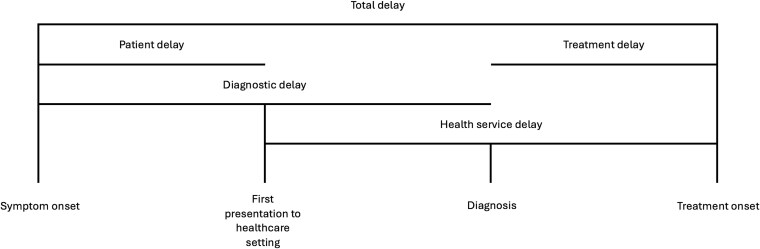
Diagram of the patient journey from symptom onset to treatment, describing the different categories of delays. Adapted from Bello *et al*.^1^

Brief details from the included studies were systematically extracted noting bibliographic details; characteristics of the study (design, sample size, and population); key findings relating reasons for delay; and where these occurred. Data extraction was undertaken independently by two authors, with a third author reviewing. Discrepancies in results were discussed with two further authors. We used an adapted version of the patient pathway of Bello *et al*.^1^ to map and synthesize data at different points of delay from symptoms to initiation of treatment, with our definitions of the different categories of delay presented in Fig. 1. We used tables to summarize evidence relating to associations between factors and delay and compiled a narrative synthesis of studies mapped to the patient pathway. Qualitative findings were synthesized to identify agreement or disagreement with quantitative studies.

### Robustness and relevance assessment

Given the rapid nature of the review, we did not carry out a formal quality assessment of individual studies but assessed each study in terms of (i) robustness (the hierarchy of study design and size of sample) and (ii) relevance (undertaken in a UK context and whether related to a whole population or a particular patient type or population sub-group). Our criteria for ranking the studies are presented in Table 2. These criteria were agreed by discussion between authors. Robustness and relevance were assessed by one reviewer and checked by a second, with any disagreements being resolved by discussion. Our criteria for ranking the studies are presented in Table 2.

**Table 2:** Relevance and robustness criteria.

Robustness		Relevance to the UK	
1	Systematic review	1	Similar incidence country, whole population
2	Cohort study, multiple institutions	2	Similar incidence country, specific population or sub-group
3	Cohort study, single institution	3	Less similar incidence country, whole population
4	Survey, multiple institutions	4	Less similar incidence country, population sub-group
5	Survey, single institution		

## Results

### Included studies

**Figure 2:**
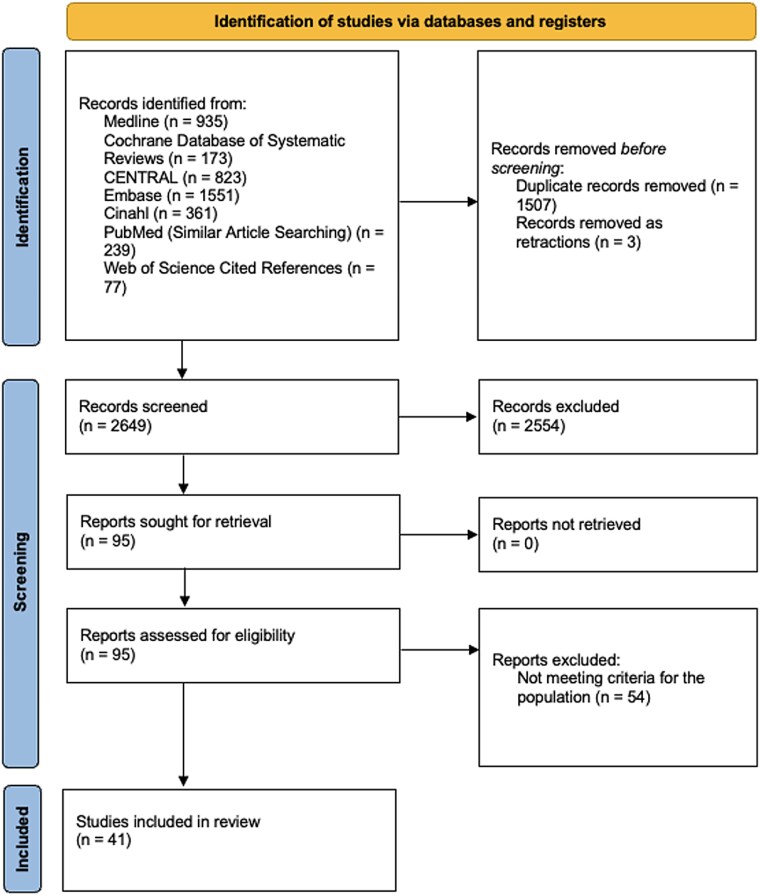
Flowchart of literature search process.

The search process using both databases and other sources is documented in Fig. 2. Thirty-seven articles from the initial search met the inclusion criteria. The database searches were updated in January 2025 yielding four additional citations for inclusion.^8–11^ Forty-one studies in total were included in the review.

### Characteristics of included evidence

Of the 41 included documents we identified 3 existing reviews, 5 qualitative studies, 18 cohort studies (reported in 19 papers), and 13 cross-sectional studies (reported in 14 papers). Studies were undertaken in 16 low-burden countries; Portugal (*n* = 8) and the UK (*n* = 6) had the highest number of studies. Most studies referred to active TB without further specification, eight related to pulmonary TB. No studies were carried out exclusively in patients with extrapulmonary TB.

### Robustness and relevance of included evidence

We identified studies that had greater or lesser robustness, and greater or lesser relevance to a UK context, with a grading of 1 being greatest. Twenty-two studies were rated as grade 1 or grade 2 for robustness (bold text), and 13 studies were rated as grade 1 for relevance (underlined).

### Classifying delay

Based on the conceptual framework of delays in diagnosis and treatment of pulmonary TB^1^ we mapped the evidence base from low-burden settings onto the framework in terms of (i) where delays can occur and (ii) what factors are associated with delays. Delays in the patient journey were mapped from symptom onset to treatment initiation.

### Synthesis—What causes delays in the patient journey from symptom onset to treatment initiation in low-burden settings?

Table 3 presents factors associated with patient delay, including whether each factor was linked to increased or reduced risk of delay, along with references to the studies that identified them. We focused on patient delay as most of the extracted results related to this stage of the diagnostic pathway. A summary of factors linked to other types of delay is provided in a table in the Supplementary material.

**Table 3:** Factors associated with patient delay.

	Increased delay	No effect on delay	Reduced delay
All patient factors		Auer^12^	
Gender	Female (**Evenden**^13^) Male (Saldana^14^)	**Dale,** ^15^ **Peri,**^16^ Türkkani,^17^ Vigneswaran,^11^ Jurcev-Savicevic,^18^ Pezzotti,^19^ **Roberts,**^20^ Stjepanovic,^21^ Williams^22^	Male with EPTB (**Dale**)^15^
Age	Age below 65 (**Chakma**^23^) Increasing age (**Evenden,**^13^ **Peri**^16^) Aged 46 and above (Salinas^24^)	Türkkani,^17^ Vigneswaran,^11^ Jurcev-Savicevic,^18^ Mor,^25^ Pezzotti,^19^ Stjepanovic,^21^ Williams^22^	Aged 14 or below (**Roberts,**^20^ Saldana^14^) Aged 15–24, or 65 and above (**Santos**^26^)
Socio-economic factors	Unemployed (**Santos**^26^) Lack of formal education (Salinas^24^)	School degree, occupation, annual income, housing conditions (**Peri**^16^) Literacy (Türkkani^17^) Employed (Mor,^25^ Williams^22^) Presence of social risk factors, or deprivation level (**Roberts**^20^) Education, employment, and socio-economic status (Stjepanovic^21^)	Higher education (Jurcev-Savicevic^18^)
Personal characteristics	Married (Bojovic^27^) White versus Asian ethnicity (**Evenden**^13^) Previous imprisonment (**Evenden**^13^) Language barriers (**Roberts**^20^)	Ethnicity (**Roberts,**^20^ Saldana^14^) Marital status (Stjepanovic^21^) English spoken at home (Williams^22^)	
Residency	Non-insured non-national Migrants (Mor^25^) Those living shorter time in country (**Peri**^16^) From a high TB incidence country (**Santos**^26^) From a low incidence country (Williams^22^) Foreign-born (**Zão**^28^)	Nationality (**Peri**^16^) Foreign-born (**Quattrocchi,**^29^ Saldana^14^) Indigenous versus non-indigenous or overseas-born Australian (Vigneswaran^11^) Time resident in UK (**Roberts**^20^) Patient residence (Türkkani^17^) Homeless (**Zão**^28^)	Being born in country (Williams^22^) Being born outside country (Pezzotti^19^) Longer period since migration (Williams^22^)
Locality	Low incidence area (**Santos**^26^)	High TB prevalence area (Mor^25^) Rurality (**Roberts,**^20^ Stjepanovic,^21^ **Zão,**^28^ Vigneswaran^11^)	
Medical history	Any comorbidity (Mor^25^) Alcohol misuse (**Evenden,**^13^ **Santos**^26^, Stjepanovic^21^) Alcohol/drug addiction (**Zão**^28^) Smoker (Mor,^25^ Jurcev-Savicevic,^18^ Türkkani,^17^ Williams^22^) No family history of TB (Stjepanovic^21^) Mental health barriers (**Roberts**^20^) History of chronic disease (**Quattrocchi**^29^) Malnutrition (Salinas^24^) Chronic renal failure, HIV infection (**Santos**^26^) Diabetes mellitus (Williams^22^)	Any comorbidity (Bojovic,^27^ **Peri**^16^) History of potential exposure (**Peri**^16^) Physical health comorbidity (**Roberts**^20^) Prior TB diagnosis (**Roberts**^20^) Chronic respiratory disease (Türkkani^17^) Asthma/COPD, chronic liver disease, chronic kidney disease, hazardous alcohol intake (Williams^22^)	History of respiratory disease (**Santos**^26^) HIV infection, non-pulmonary co-morbidities (**Zão**^28^)

(*Continued*)

**Table 3:** Continued

	Increased delay	No effect on delay	Reduced delay
Health behaviour	Negative attitude to TB (Bojovic^27^) Neglect of symptoms (Morais^10^) Low priority given to health (Kato^30^) Disliking hospital visits (Kato^30^) Fear of consequences (**Peri**^16^) Taking over-the-counter medication (Kato^30^) Stigma (**Quattrocchi**^29^)	Underestimating symptoms, mild nature (**Peri**^16^) Thinks they have TB (**Quattrocchi**^29^)	
Health literacy		Incorrect beliefs, poor knowledge of TB **(Peri,**^16^ **Quattrocchi**^29^)	Greater knowledge of TB (Bojovic^27^)
Availability of healthcare	Living further from health unit (Salinas^24^) Close distance to first medical visit (**Quattrocchi**^29^) Paying for transport and distance to health centre (**Quattrocchi**^29^) Lack of family physician (Kato^30^) Lack of consultation (Kato^30^) Consultation with private physician (Salinas^24^)	First assessment by GP (**Peri**^16^) Barriers to healthcare access (**Peri**^16^)	Being followed up by GP (**Tattevin**^31^) Greater distance to healthcare (**Zão**^28^)
Symptoms	Being asymptomatic (Mor^25^) Productive of sputum and haemoptysis (Kato^30^) Fever (Pezzotti^19^) Unintentional weight loss (Pezzotti,^19^ **Quattrocchi**^29^) Tiredness/weakness (**Quattrocchi**^29^) Chest pain (**Quattrocchi**^29^) Cough for 2 weeks or longer, chest pain (Salinas^24^)	Presence of symptoms (Stjepanovic^21^) Absence of respiratory symptoms (**Peri**^16^) Cough, haemoptysis, night sweats, chest pain, dyspnoea, respiratory symptoms, non-respiratory symptoms (Pezzotti^19^) Night sweats, malaise (Salinas^24^) Cough, haemoptysis, chest pain, sweats, fevers, or dyspnoea (Williams^22^)	Patient experiencing a fever (**Tattevin**^31^) Cough (Jurcev-Savicevic^18^) Losing weight (Jurcev-Savicevic^18^) Symptoms including haemoptysis, fever, dyspnoea, or chest pain (Türkkani^17^) Fatigue (Williams^22^)
Type of TB	Extra-pulmonary TB (Vigneswaran,^11^ **Loutet,**^32^ Pezzotti^19^) Smear negative (Vigneswaran^11^)	Extra-pulmonary TB (**Peri,**^16^ Saldana^14^) Patient TB category (Stjepanovic^21^) Drug susceptible (Vigneswaran^11^) CXR grade (Vigneswaran^11^) Cavitations on chest radiography (Mor^25^) Sputum or culture positive/negative (Saldana^14^)	

Studies in bold were graded as having the greatest relevance (score one).

Studies underlined were graded as having higher robustness (score one or two).

### What factors are associated with patient delay?

Patient delay describes the delay in patients seeking healthcare once they are symptomatic. The evidence on whether there are any predictors of patient delay is mixed and contradictory—our findings mirror those of Auer *et al*.^12^ Many of our included studies examined patient characteristics and their relationship with delay. There was no evidence of a clear relationship between patient delay and patient age, nor between patient delay and patient gender. There was also contradictory evidence relating to socio-economic factors, unemployment, poor literacy, and economic constraints. Evidence looking at associations between delay and residency (nine studies) were mixed, with reports of longer and shorter periods of residency and recent arrival from either a high or low incidence country being associated with patient delay. Evidence on the association between medical history and patient delay was more consistent. Studies found that delay was greater in patients with a dependency on drugs, alcohol or tobacco, or no family history of TB. The evidence was also supportive of an association between longer patient delay and reluctance to seek health care, fear regarding stigma or consequences (of a TB diagnosis), low prioritization of health (over other competing concerns) and preference for taking over the counter medication. Reduced access to healthcare was also associated with patient delay with specific associations between distance and delay and availability and delay. Evidence regarding the relationship between symptoms and patient delay was contradictory and inconclusive.

### What factors are associated with diagnostic delay?

Evidence relating to delays in diagnosis following first contact with healthcare professionals indicated that contact in health locations other than primary care was associated with less delay. A smear negative sputum test, and use of a trial of empirical antibiotic treatment (for non-tuberculous causes) were associated with greater delay in diagnosis. In general, older patients, and female patients, appeared at higher risk of diagnostic delay. Factors associated with lower socio-economic status, such as not completing high school or unemployment, were associated with increased delays.

### What factors are associated with treatment delay?

None of the studies reviewed presented results on factors associated with treatment delay as per our definition (days from diagnosis to treatment initiation). This likely reflects the established practice in many of these higher-income, lower-TB burden settings, to initiate TB treatment rapidly after diagnosis.

### What factors are associated with health service delay?

There was a general association between longer health service delays and patients’ healthcare contact being in general practice/outpatient rather than inpatient/emergency department (ED) or specialist care. Being treated for other conditions or having a negative sputum smear were associated with greater delay, while greater access to relevant diagnostic imaging was associated with a lesser delay. Evidence from three studies (including one of greater robustness and two of greater relevance) was consistent that extra-pulmonary TB was associated with greater delay. Evidence of associations between age or gender and delays were mixed, although the bulk of included evidence did support an association between being female and health service delays. Two studies identified that patients having pre-existing co-morbidity, including respiratory disease, was associated with greater healthcare delay. Reports of an association between residential status and delay were mixed, with no clear pattern.

### What factors are associated with total delay?

Relatively few studies reported outcomes on total delay. There was strong and consistent evidence that extra-pulmonary TB was associated with greater delay. The evidence on age and gender was mixed. Similarly, multiple studies found no association between smear status and delays, while one study did find an association between being smear negative and increased total delay.

### Qualitative evidence on factors associated with delay

Studies using qualitative methods supported the evidence from quantitative studies that access to healthcare, patient health behaviours, and healthcare practitioner index of suspicion for TB contributed to delays. Patients described how lack of easy access to primary care and/or use of private services had delayed their diagnosis. One study highlighted how limited knowledge of services could make ED attendance the preferred option.^33^ Again, initial access via primary care rather than secondary or specialist care was cited as a source of delay. In contrast, screening programmes for new arrivals to a country were described as helping to reduce delay.

Reports of health behaviours increasing delay were similar to the quantitative literature, with a low perceived risk or a perception that illness was not severe, fears of the consequences, and perceived stigma of TB associated with delay. Knowing other people who had been diagnosed with TB could encourage a patient to seek help when experiencing similar symptoms. While our included quantitative studies suggested that limited patient knowledge of TB causes and treatability was associated with increased delay, a review of qualitative studies that focused on migrants found that knowledge of classical TB symptoms tended to be good in these groups but knowledge of treatability was poor.^34^ One qualitative study of doctors working with prison populations did highlight low health literacy was a perceived barrier to TB diagnosis.^8^ Reports from patients and practitioners also highlighted the challenges of atypical, extrapulmonary, or non-specific symptoms, with some community staff noting that they saw very few TB cases. There was a suggestion that symptoms of TB were not always well known to practitioners, and that clinical suspicion of the disease could often be low. Patients felt their symptoms were not taken seriously and were frustrated by absent or delayed referrals for diagnosis and specialist review. Taking a full history from patients who had limited English was described as challenging in an English-speaking setting.

## Discussion

### Main findings of this study

We identified 41 separate studies of relevance to the UK low-burden TB setting that reported factors associated with delays at different points in the TB patient’s pathway. Patient centred factors were related to health status, health behaviours, and knowledge. Factors associated with diagnostic delay were service location (primary care versus secondary), low physician suspicion for TB, issues with accuracy and timing of testing, and patients who were older or female. Studies that examined total delay identified associations with delay among patients with extrapulmonary TB. The evidence of the impact of age or gender on total delay was mixed and unclear.

The evidence base varied in robustness of findings, the degree of relevance to the UK, and the direction of effect for some factors. The mixed direction of findings likely reflects heterogeneity in populations and methodologies between studies. Most studies investigated individual patient characteristics. A smaller number considered delays relating to individual healthcare providers, but there were few studies presenting evidence on how the whole health system or, more specifically, TB service pathways, were associated with delays.

### What is already known on this topic

Interventions and strategies to reduce delays in seeking diagnosis and receiving treatment will have a beneficial effect on the transmission of TB which will lead to improved patient and health service outcomes.^13^ The evidence base so far has focused on higher burden settings and on calculating delays. While many of the factors we have found are also associated with delays in high-burden settings (e.g. older age, female, atypical presenting symptoms), their relative importance is arguably lesser in these higher burden settings in which the removal of more basic barriers to healthcare access are a higher priority for public health.^2^

### What this study adds

This work has identified several patient and health service factors that are consistently associated with patient, diagnostic, and total delay from TB symptom onset to initiation of treatment in low-burden settings relevant to the UK. While none of the studies tested causality, concordant findings from qualitative studies suggest that this may be likely. Those factors, particularly on the health service side, that could be amenable to change should now be the focus of interventional studies aimed at reducing delays in TB diagnosis and treatment. Health service factors amenable to change may include improving clinical awareness of the atypical presenting symptoms of pulmonary and extrapulmonary TB, particularly in primary care/outpatient settings, and targeting reductions in delays at population groups at greater risk: older adults and females.

### Limitations of this study

There was limited qualitative evidence and it would be helpful for further qualitative exploration of the associations between factors and delay, particularly given that some studies were in highly selected populations (specific ethnic groups, homeless with complex needs, prisons). Furthermore, heterogeneity between study settings and methodologies precluded attempts at quantitatively synthesizing the outcomes reported. Future reviews with narrower inclusion criteria may reduce heterogeneity between included studies, allowing for reasonable quantitative synthesis. This work was carried out in 3 months using rapid review methods. As with any review, there is potential that relevant literature was not identified during our searching processes. However, we believe that our use of supplementary searching in addition to electronic databases, together with multiple team members who carried out duplicate checking of retrieved citations will have added to the robustness of the review. We acknowledge that our rating of robustness and relevance has considerable limitations and does not provide a detailed assessment of quality, however, the categorizations provide a useful indicator to aid understanding of the evidence.

In this review we applied a definition of a low-burden TB setting as a country with an incidence rate of < 40 per 100,000. Had we applied the WHO definition of < 10 per 100,000 we would have excluded studies in Portugal (*n* = 8), Turkey (*n* = 2), and one study each from Japan, Mexico, and Serbia. Of these the majority had 2020 incidence rates close to the 10 per 100 000 threshold, although Mexico had an incidence rate of ~24 per 100 000.^4^ Exclusion of these studies produced little difference in our findings, although it did reduce some of the contrasting evidence around the association of patient symptoms with diagnostic delays.

## Supplementary Material

Supplementary_Material_fdaf106

## Data Availability

The data underlying this article are available in the article and in its Supplementary material.

## Notes

**Louise Preston,** Senior Lecturer

**Alexander Thompson,** Public Health Registrar

**Susan Baxter,** Senior Research Fellow

**Duncan Chambers,** Research Fellow

**Paul Collini,** Senior Clinical Lecturer

**Louise Falzon,** Research Associate

**Jack Goodall,** Registrar in Infectious Disease

**Andrew Lee,** Professor
