# Supplementary material for: A rapid review of the causes of diagnostic and treatment delays for tuberculosis in low-burden countries
Source: J Public Health (Oxf). 2025 Sep 12;47(4):e530–9. doi: 10.1093/pubmed/fdaf106 (PMC12669990; doi:10.1093/pubmed/fdaf106)
Supplement: Supplementary_Material_fdaf106 [file supplementary_material_fdaf106.docx]

# Supplementary Material

# Appendix One - Quantitative Evidence Table

Studies in bold were graded as having greatest relevance (score one) and studies underlined were graded as having higher robustness (score one or two).

Studies in italics are evidence reviews.

| **Pathway** | **Category** | **Increased delay** | **No effect on delay** | **Reduced delay** |
| --- | --- | --- | --- | --- |
| Diagnostic delay | Healthcare system | Weekend consultations (Miller)  Fewer health centres (Soares) |  | First presentation in outpatient, inpatient or ED settings (Miller)  Having medical insurance (**Tattevin**) |
|  | Diagnostics | Negative sputum smear (Bojovic)  Had chest x ray or CT scan in previous year (Miller)  Received fluoroquinolone in previous year (Miller)  EPTB (**Peri**) | Smear positive versus smear negative (Ekinci) | Screening using mobile chest x ray (*Heuvelings*) |
|  | Age | Age > 47 (Bojovic)  Age ≥ 65 (Miller)  Age >45 (**Santos**)  Higher old age dependency ratio (Soares) |  | Aged 5-24yrs (**Santos**) |
|  | Gender | Female (**Chakma**, **Santos**) |  | Male (**Auer**, **Nunes**) |
|  | Medical history | History of asthma or COPD (Miller) |  | HIV positive (**Nunes**, **Santos**)  Previous TB diagnosis (**Tattevin**) |
|  | Locality | Greater proportion men (Soares)  Greater proportion of immigrants (Soares)  Smaller population density (Soares) |  | Metropolitan locations (Miller) |
|  | Residency | Born in country with high TB incidence (**Santos**) | Foreign born (**Peri**) |  |
|  | Socio-economic factors | Higher levels of high school drop out (Soares)  Lower proportion unemployed (Soares) |  |  |
|  | Personal characteristics |  |  | Health professional (**Santos**) |
| Health Service Delay | Healthcare system | Initially seeking advice from a GP (Pezzotti, **Quattrocchi**)  Visit to outpatient clinic (Pezzotti)  Referred from primary care (**Roberts**)  Visited GP/Paediatrician (**Auer**, **Evenden**)  Seeing multiple physicians (**Auer**)  Delay in seeing a specialist (Bojovic)  Patient delay > 30 days (Pezzotti)  Repeated visits with different providers (**Quattrocchi**)  Treated with antibiotics (Wang) | Emergency visit (El Halabi)  Admitted to hospital (El Halabi, Mor)  Treated for CAP (El Halabi)  Treated with fluoroquinolone (El Halabi) | Seeking care at hospital (**Quattrocchi**)  Referral from secondary care (**Roberts**)  Care by a specialist TB provider (El Halabi) |
|  | Diagnostics | Lack of availability of biopsy (*Mathiasen*)  Prior TB treatment (**Quattrocchi**, Pezzotti)  Smear negativity (**Roberts**)  Delay in having a chest x-ray (Bojovic)  CXR performed in previous 6 months (Wang)  Visit by a specialist (Wang) |  | X ray at first consultation (**Auer**)  Use of chest imaging (El Halabi)  Use of tuberculosis NAATs (El Halabi) |
|  | Type of TB | Extra-pulmonary TB (Saldana), (**Zão**), (**Loutet**)  Smear negative (Ekinci, Jurcev- Savicevic, Saldana) | Extra-pulmonary TB (Vigneswaran, Pezzotti)  Drug susceptible (Vigneswaran)  Smear negative (Vigneswaran)  CXR grade (Vigneswaran)  Culture positive (Mor, Saldana) |  |
|  | Symptoms | Asymptomatic (Mor)  Cough (Pezzotti, **Quattrocchi**) | Chest pain (**Auer,** Pezzotti)  Fever, weight loss, night sweats, haemoptysis, loss of appetite, fatigue, dyspnoea, respiratory symptoms, non-respiratory symptoms (Pezzotti) | Higher number of TB symptoms (Bojovic)  Dizziness (**Quattrocchi**) |
|  | Age | Age ≥ 65 (Saldana)  Age ≥ 45 (Vigneswaran)  Older age (El Halabi, **Evenden**) | Jurcev- Savicevic, Mor, Pezzotti, **Quattrocchi**, **Roberts**, Wang |  |
|  | Gender | Female (Saldana, **Roberts**, **Evenden**, Jurcev- Savicevic) | Vigneswaran, El Halabi, Wang  If EPTB or lymphadenitis (**Dale**) | Female (**Quattrocchi**)  Males with pulmonary TB (**Dale**) |
|  | Medical history | Co-morbidities (**Zão**, Bojovic, Mor)  Non HIV immunosuppression (El Halabi)  Lung cancer, sarcoidosis, COPD (**Zão**) | Comorbid COPD (**Auer**)  HIV positive (El Halabi, **Zão**)  Diabetes (El Halabi)  Number of ICD entries at first visit (El Halabi)  Prior TB diagnosis (**Roberts**)  Physical health comorbidity (**Roberts**)  Alcohol abuse or drug abuse (Wang)  Hepatitis C positive (Wang)  Non-pulmonary comorbidities (**Zão**) | HIV positive (Wang)  Alcohol and drugs addiction (**Zão**) |
|  | Patient characteristics | White ethnicity (**Evenden**) | Ethnicity (**Roberts**, Saldana)  Foreign born (Wang)  Socio-economic status (Wang) | Increasing socioeconomic deprivation (**Zão**) |
|  | Locality | Increasing distance to healthcare (**Zão**) | Remote residence versus urban residence (Vigneswaran)  Incidence level of local area (**Roberts**) |  |
|  | Residency |  | Indigenous versus Non-indigeneous or overseas-born Australian (Vigneswaran)  Resident in a high incidence state (El Halabi)  Born in UK (Saldana)  Non-insured non-national migrants (Mor) | Born outside country (Pezzotti. **Quattrocchi**, **Zão**)  Recent residency (**Roberts**) |
| Total delay | Access to healthcare | Paying for transportation, distance to health centre (**Quattrocchi**)  Close distance to first visit place (**Quattrocchi**) | No health insurance (Han) | Repeated visits with the same provider (**Quattrocchi**) |
|  | Diagnostics | Prior TB treatment (**Quattrocchi**) | Sputum submitted at first consultation (**Auer**) | X-ray examination at first consultation (**Auer**) |
|  | Symptoms |  |  | Haemoptysis, cough > 3 weeks, weight loss (**Quattrocchi**) |
|  | Type of TB | Extra-pulmonary TB (**Loutet**, Saldana, Vigneswaran)  Smear negative (Vigneswaran) | Drug susceptible (Vigneswaran)  CXR grade (Vigneswaran)  Smear positive, cavitary, or disseminated pulmonary TB (Han)  Smear status, culture status (Saldana) |  |
|  | Gender | Female (**Loutet**) | Vigneswaran, Han, Saldana |  |
|  | Age | Aged ≥ 45 (**Loutet**)  Older age (Saldana) | Vigneswaran, Han |  |
|  | Medical history |  | Number of underlying medical conditions (Han)  Previous diagnosis (**Loutet**) |  |
|  | Patient characteristics |  | Hispanic or Latino Ethnicity (Han)  First-language not English (Han)  Essential worker (Han) | Black other ethnicity compared to Bangladesh (Saldana) |
|  | Health literacy | Knowledge of TB as infectious disease (**Quattrocchi**) |  |  |
|  | Locality | Living outside London (**Loutet**) | Remote residence versus urban residence (Vigneswaran) |  |
|  | Residency | Recent migrant to UK (**Loutet**) | Indigenous versus Non-indigeneous or overseas-born Australian (Vigneswaran)  Foreign-born (**Quattrocchi**) |  |

# Appendix Two - Qualitative Evidence Table

| **Pathway** | **Category** | **Increased delay** | **No effect** | **Reduced Delay** |
| --- | --- | --- | --- | --- |
| Patient | Access to healthcare | Difficulties in access (***De Vries***)  Let down by system (***De Vries***)  Language barriers (***De Vries***) | Patients perceive that they present soon after symptoms (Gerrish) | Low perceived barriers to access (Ribero)  Trust in healthcare system (Ribero) |
|  | Symptoms | Symptoms unspecific (Ribero)  Mild intermittent symptoms (Sagbakken)  Cough, but no other symptoms (Sagbakken) |  |  |
|  | Health behaviour | Practitioners perceived late presentation due to stigma (Gerrish)  Perceived benefits of seeking healthcare low (Ribero)  Perceived low severity of disease (Ribero)  Perceived limited likelihood of having illness (Sagbakken)  Drug users concerned about forced abstinence (Craig)  Lack of trust in practitioners, negative attitudes, fear of testing (***De Vries***)  Stigma (***De Vries***) |  | High perceived severity of disease (Ribero)  Strong cue to action (Ribero) |
|  | Health literacy | Knowledge of TB as only as a lung disease, perception of TB as only linked with poor diet and poverty (Sagbakken)  Limited perception of susceptibility, causes and transmission, knowledge of symptoms good, views on treatability often inaccurate (***De Vries***)  Awareness of personal risk low (Craig)  Symptoms not recognised, attributed to other causes, accessed healthcare for other reasons (Craig)  Low awareness of personal risk of TB (Craig)  Low level of knowledge about TB among prison inmates (Aguiar) |  | Satisfaction, familiarity, accessibility, knowledge of the service, literacy and cultural factors associated with preference for ED (Sagbakken)  Knowledge of symptoms in others (Sagbakken) |
|  | Other patient characteristics |  | Residents and migrants similar behaviour patterns (Ribero) |  |
| Total health service delay | Access to healthcare | Patients perceived symptoms not treated seriously (Gerrish)  Patients perceived slow referral when symptoms persisted, frustration at time took for diagnosis to be confirmed (Gerrish)  Patients reported repeat visits to GP, prescribed analgesia/antibiotics (Gerrish)  Patients accessing healthcare privately (Ribero)  1^st^ visit to GP (Ribero)  Lack of primary care service (Ribero)  Problems with care co-ordination, lack of specialists (***De Vries***) |  | Perception amongst practitioners that GPs were becoming more aware (Gerrish)  TB screening programme (Ribero)  Being screening for TB on arrival to a new country (Sagbakken)  Specialist consultation (Ribero) |
|  | Symptoms | Atypical symptoms made diagnosis challenging (Gerrish)  Typically long deterioration, unspecific symptoms (Ribero)  Atypical presentation, limited number of cases seen (***De Vries***) |  | Medical history giving suspicion of TB (Sagbakken)  Presenting with typical TB symptoms (Sagbakken) |
|  | Clinical suspicion of TB | Low clinical suspicion of TB amongst GPs (Gerrish)  Diagnosis harder if few cases seen (Gerrish)  Health professionals not associating patient presentation with TB, delay initiating tests (Sagbakken)  Lack of awareness of symptoms (typical and atypical) amongst healthcare professionals (Sagbakken) |  |  |
|  | Testing | Few patients referred for chest X-ray or sputum test on first presentation (Sagbakken) |  |  |
|  | Patient-related factors | Patients without English as first language could make history-taking challenging (Gerrish) |  |  |
|  | Type of TB | EPTB (Ribero)  Delay in testing for EPTB(Sagbakken)  EPTB diffuse and non-specific symptoms (Sagbakken) |  |  |

# Appendix Three - Included Studies (*) and other references

1. *Aguiar A, Abreu M, Duarte R. Healthcare professionals perspectives on tuberculosis barriers in Portuguese prisons - a qualitative study. *J Public Health (Oxf).* 2024;46(3):e389-399
2. * Auer C, Kiefer S, Zuske M. et al. Health-seeking behaviour and treatment delay in patients with pulmonary tuberculosis in Switzerland: some slip through the net. *Swiss Med Wkly* 2018;148:w14659.
3. Bello S, Afolabi R, Ajayi D. Empirical evidence of delays in diagnosis and treatment of pulmonary tuberculosis: systematic review and meta-regression analysis. *BMC Public Health* 2019;19(1):1-11.
4. *Bojovic O, Medenica M, Zivkovic D. et al. Factors associated with patient and health system delays in diagnosis and treatment of tuberculosis in Montenegro, 2015-2016. *PLoS ONE* 2018;13(3):e0193997.
5. *Chakma B, Gomes D, Filipe P. et al. A temporal analysis on patient and health service delays in pulmonary tuberculosis in Portugal: inter and intra-regional differences and in(equalities) between gender and age. *BMC Public Health* 2022;22(1):1830.
6. *Craig GM, Joly LM, Zumla A. 'Complex' but coping: experience of symptoms of tuberculosis and health care seeking behaviours--a qualitative interview study of urban risk groups, London, UK. *BMC Public Health.* 2014;14:618.
7. *Dale K, Tay E, Trauer J, Trevan P, Denholm J. Gender differences in tuberculosis diagnosis, treatment and outcomes in Victoria, Australia, 2002-2015. *Int J Tuberc Lung Dis.* 2017;21(12):1264-1271.
8. *de Vries S, Cremers A, Heuvelings C. et al. Barriers and facilitators to the uptake of tuberculosis diagnostic and treatment services by hard-to-reach populations in countries of low and medium tuberculosis incidence: a systematic review of qualitative literature. *Lancet Infect Dis.* 2017;17(5):E128-E143.
9. *Ekinci G, Karakaya E, Ongel E, Haciomeroglu O, Yilmaz A. Patient and doctor delays in smear-negative and smear-positive pulmonary tuberculosis patients attending a referral hospital in Istanbul, Turkey. *ScientificWorldJournal.* 2014;158186.
10. *El Halabi J, Palmer N, McDuffie M. et al. Measuring health-care delays among privately insured patients with tuberculosis in the USA: an observational cohort study. *Lancet Infect Dis.* 2021;21(8):1175-1183.
11. *Evenden P, Roche A, Karo B, Balasegaram S, Anderson C. Presentation and healthcare delays among people with tuberculosis in London, and the impact on treatment outcome. *BMJ Open Respir Res.* 2019;6(1):e000468.
12. *Han E, Nabity S, Dasgupta-Tsinikas S. et al. Tuberculosis Diagnostic Delays and Treatment Outcomes among Patients with COVID-19, California, USA, 2020. *Emerg Infect Dis* 2024;30(1):136-140
13. *Kato Y, Arimoto A, Shimamura T, Murashima S. [Factors associated with delay in seeking medical treatment in pulmonary tuberculosis patients in Japan]. *Nihon Koshu Eisei Zasshi.* 2012;59(4):251-258.
14. *Gerrish K, Naisby A, Ismail M. Experiences of the diagnosis and management of tuberculosis: a focused ethnography of Somali patients and healthcare professionals in the UK. *J Adv Nurs.* 2013;69(10):2285-94.
15. *Heuvelings C, de Vries S, Greve P et al. Effectiveness of interventions for diagnosis and treatment of tuberculosis in hard-to-reach populations in countries of low and medium tuberculosis incidence: a systematic review. *Lancet Infect Dis.* 2017;17(5):e144-e58.
16. *Jurcev-Savicevic A, Kardum G. Health-care seeking behaviour for tuberculosis symptoms in Croatia. *Eur J Public Health.* 2012;22(4):573-7.
17. *Jurcev-Savicevic A, Mulic R, Kozul K. et al. Health system delay in pulmonary tuberculosis treatment in a country with an intermediate burden of tuberculosis: a cross-sectional study. *BMC Public Health.* 2013;13:250.
18. *Loutet MG, Sinclair C, Whitehead N, Cosgrove C, Lalor MK, Thomas HL. Delay from symptom onset to treatment start among tuberculosis patients in England, 2012-2015. *Epidemiol Infect.* 2018;146(12):1511-8.
19. *Mathiasen Victor D, Wejse C, Lillebaek T, Hansen Anders K, Eiset Andreas H. Delays in the Diagnosis and Treatment of Tuberculous Lymphadenitis in Low-Incidence Countries: A Systematic Review. *Respiration.* 2019;97(6):576-84.
20. *Miller A, Arakkal A, Koeneman S. et al. Incidence, duration and risk factors associated with delayed and missed diagnostic opportunities related to tuberculosis: a population-based longitudinal study. *BMJ Open* 2021;11(2):e045605.
21. *Morais M, Sousa S, Marques J. et al. Investigating the role of symptom valorisation in tuberculosis patient delay in urban areas in Portugal. *BMC Public Health* 2023;23(1):2421
22. *Mor Z, Kolb H, Lidji M, Migliori G, Leventhal A. Tuberculosis diagnostic delay and therapy outcomes of non-national migrants in Tel Aviv, 1998-2008. *Euro Surveill.* 2013;18(12):21.
23. *Nunes C. and Taylor B. Modelling the time to detection of urban tuberculosis in two big cities in Portugal: a spatial survival analysis. *Int J TubercLung Dis.* 2016;20(9):1219-1225.
24. *Peri A, Bernasconi D, Galizzi N. et al. Determinants of patient and health care services delays for tuberculosis diagnosis in Italy: a cross-sectional observational study. *BMC Infect Dis.* 2018;18(1):690.
25. *Pezzotti P, Pozzato S, Ferroni E, et al. Delay in diagnosis of pulmonary tuberculosis: a survey in the Lazio region, Italy. *Epidemiol Biostat Public Health.* 2015;12:1-10.
26. *Quattrocchi A, Barchitta M, Nobile C. et al. Determinants of patient and health system delay among Italian and foreign-born patients with pulmonary tuberculosis: a multicentre cross-sectional study. *BMJ Open* 2018;8(8):e019673.
27. *Ribeiro RM, Havik PJ, Craveiro I. The circuits of healthcare: Understanding healthcare seeking behaviour-A qualitative study with tuberculosis patients in Lisbon, Portugal. *PLoS One*. 2021;16(12):e0261688.
28. *Roberts D, Mannes T, Verlander N, Anderson C. Factors associated with delay in treatment initiation for pulmonary tuberculosis. *ERJ Open Res* 2020;6(1).
29. *Sagbakken M, Bjune G, and Frich J. Experiences of being diagnosed with tuberculosis among immigrants in Norway--factors associated with diagnostic delay: a qualitative study. *Scand J Public Health* 2010;38(3):283-290.
30. *Saldana L, Abid M, McCarthy N, Hunter N, Inglis R, Anders K. Factors affecting delay in initiation of treatment of tuberculosis in the Thames Valley, UK. *Public Health* 2013;127(2):171-177.
31. *Salinas J, Calvillo S, Cayla J, Nedel F, Martin M, Navarro A. Delays in the diagnosis of pulmonary tuberculosis in Coahuila, Mexico. *Int J Tuberc Lung Dis.* 2012;16(9):1193-1198.
32. *Santos J, Soares P, Leite A, Duarte R, Nunes C. Patient and healthcare delays in critical and non-critical pulmonary tuberculosis incidence areas in Portugal: are there differences? *Public Health.* 2021;201:41-47.
33. *Santos J, Leite A, Soares P, Duarte R, Nunes C. Delayed diagnosis of active pulmonary tuberculosis - potential risk factors for patient and healthcare delays in Portugal. *BMC Public Health* 2021;21(1):2178.
34. *Soares P, Aguiar A, Leite A, Duarte R, Nunes C. Ecological factors associated with areas of high tuberculosis diagnosis delay. *Public Health*. 2022;208:32-9.
35. *Stjepanovic M, Skodric-Trifunovic V, Radisavljevic-Pavlovic S. et al. Patient, Healthcare System and Total Delay in Tuberculosis Diagnosis and Treatment Among Serbian Population. *Acta Clin Croat* 2018;57(2):257-263.
36. *Tattevin P, Che D, Fraisse P. et al. Factors associated with patient and health care system delay in the diagnosis of tuberculosis in France. *Int JTuberc Lung Dis.* 2012;16(4):510-515.
37. *Türkkani M, Özdemir T, and Özdilekcan Ç. Determination of related factors about diagnostic and treatment delays in patients with smear-positive pulmonary tuberculosis in Turkey. *Turk J Med Sci* 2020;50(5):1371-1379.
38. *Vigneswaran N, Parnis R, Lowbridge C, Townsend D, Ralph A. Factors leading to diagnostic delay in tuberculosis in the tropical north of Australia. *Intern Med J 2024;*54(4):582-587
39. *Wang M, Fitzgerald J, Richardson K. et al. Is the delay in diagnosis of pulmonary tuberculosis related to exposure to fluoroquinolones or any antibiotic? *Int J Tuberc Lung Dis.* 2011;15(8):1062-1068.
40. *Williams E, Cheng A, Lane G, Guy S. Delays in presentation and diagnosis of pulmonary tuberculosis: a retrospective study of a tertiary health service in Western Melbourne, 2011-2014. *Intern Med J* 2018;48(2):184-193.
41. * Zão I, Ribeiro A, Apolinario D, Duarte R. Why does it take so long? The reasons behind tuberculosis treatment delay in Portugal. *Pulmonology* 2019;25(4):215-222.

# Appendix Four - World Health Organisation (WHO) [list](https://www.gov.uk/government/publications/tuberculosis-tb-by-country-rates-per-100000-people/who-estimates-of-tuberculosis-incidence-by-country-and-territory-2020-accessible-text-version#overview) of low incidence TB countries

| Albania  American Samoa  Andorra  Anguilla  Antigua and Barbuda  Argentina  Armenia  Aruba  Australia  Austria  Bahamas  Bahrain  Barbados  Belarus  Belgium  Belize  Bermuda  Bosnia and Herzegovina  British Virgin Islands  Bulgaria  Cabo Verde  Canada  Cayman Islands  Chile  Colombia  Comoros  Cook Islands  Costa Rica  Croatia  Cuba  CuraÃ§ao  Cyprus  Czechia  Denmark  Dominica  Egypt  Estonia | Finland  France  French Polynesia  Germany  Greece  Grenada  Guam  Guatemala  Honduras  Hungary  Iceland  Iran  Iraq  Ireland  Israel  Italy  Jamaica  Japan  Jordan  Kuwait  Latvia  Lebanon  Lithuania  Luxembourg  Maldives  Malta  Mauritius  Mexico  Monaco  Montenegro  Montserrat  Netherlands  New Caledonia  New Zealand  Niue  North Macedonia  Norway | Oman  Palau  Panama  Poland  Portugal  Puerto Rico  Qatar  Saint Kitts and Nevis  Saint Lucia  Saint Vincent and the Grenadines  Samoa  San Marino  Saudi Arabia  Serbia  Seychelles  Sint Maarten  Slovakia  Slovenia  Spain  Suriname  Sweden  Switzerland  Syrian Arab Republic  Togo  Tonga  Trinidad and Tobago  Tunisia  Turkey  Turks and Caicos Islands  United Arab Emirates  United Kingdom  United States of America  Uruguay  Vanuatu  Wallis and Futuna Islands  Occupied Palestinian territory, including east Jerusalem |
| --- | --- | --- |

# Appendix Five- Search strategy

| MEDLINE  1 exp Tuberculosis/  2 (tuberculosis or tb).tw.  3 1 or 2  4 Delayed Diagnosis/  5 (delay* adj3 diagnos*).tw.  6 4 or 5  7 symptom*.tw.  8 and/3,6-7  9 exp animals/ not humans.sh.  10 8 not 9 | Embase  1 exp tuberculosis/  2 (tuberculosis or tb).tw.  3 1 or 2  4 delayed diagnosis/  5 (delay* adj3 diagnos*).tw.  6 4 or 5  7 symptom*.tw.  8 and/3,6-7  9 limit 8 to human | The Cochrane Library (Cochrane Database of Systematic Reviews and CENTRAL)  #1 MeSH descriptor: [Tuberculosis] explode all trees  #2 (tuberculosis or tb):ti,ab  #3 #1 OR #2  #4 MeSH descriptor: [Delayed Diagnosis] this term only  #5 (delay* NEAR/3 diagnos*):ti,ab  #6 4 or 5  #7 symptom*:ti,ab  #8 #3 AND #6 AND #7 | CINAHL  S1 (MH "Tuberculosis+")  S2 TI ( tuberculosis OR tb ) OR AB ( tuberculosis OR tb )  S3 S1 OR S2  S4 (MH "Diagnosis, Delayed")  S5 TI delay* N3 diagnos* OR AB delay* N3 diagnos*  S6 S4 OR S5  S7 S3 AND S6 Limiters Human |
| --- | --- | --- | --- |

# Appendix Six - Data Tables

## Extraction Table 1: Reviews

| First author, date  Countries included  Number of studies | Date inclusion, design inclusion, population inclusion  Study design inclusion | Main findings (points in pathway, main obstacles) | Comments re relevance/ robustness |
| --- | --- | --- | --- |
| De Vries, 2017  12 studies | 1990 onwards.  Hard to reach populations, OECD countries  Qualitative studies | Patient delay – limited perception of susceptibility, causes and transmission, knowledge of symptoms good, views on treatability often inaccurate. Difficulties in access reported, feeling of being let down by system not trusting practitioners, negative attitudes or fear prevented people getting tested, concerns regarding loss of privacy/lack of confidentiality. Stigma was described in all included articles. Service providers described atypical presentation of the disease due to different cultural perspectives, language barriers (and the lack of  professional translators), paucity of TB cases seen per year, and patient delay contributing to diagnostic delay. Problems in co-ordinated care and lack of specialists also reported. | 4 included studies from the UK. |
| Heuvlings, 2017  19 studies | 1990 onwards.  TB cases  Hard to reach populations  Interventions to improve diagnosis and treatment  Qualitative or quantitative, cost effectiveness | Healthcare system - Screening by (mobile) chest radiography improved coverage, identification, and reduced diagnostic delay. It is more cost-effective among migrant and homeless populations than TB skin test.  Sputum culture for pre-migration screening and active referral to a TB clinic improved identification. | Only for hard to reach populations, doesn’t include studies of barriers, only looks at interventional studies. |
| Mathiasen, 2019  11 studies included | Low incidence countries  TB  Studies reporting on delay  Tuberculous lymphadenitis  Not case reports or reviews | Patient delay - a fluctuating neck mass or discharging sinus (n = 13) was associated with a delay of 196 days. African and Afro-Caribbean patients had longer mean delays compared with Asians: 196 (range, 35–238) versus 98 days (range, 21–406).  Healthcare delay - patients with a neck lump were referred to one of seven specialities. Patients had an average of four hospital appointments (range, 3–6) including the department where relevant treatment was initiated. The greatest contribution to delay was between the first outpatient consultation and biopsy of the lymph node with a mean delay of 43 days (range, 1–153). | Excludes PTB and only considers tuberculous lymphadenitis |

## Extraction Table 2: quantitative studies

| First author, date  Country of origin | Study design Sample size  Population  Pulmonary or extrapulmonary | Main findings (points in pathway, main obstacles) | Comments re relevance /robustness |
| --- | --- | --- | --- |
| Auer, 2018  Switzerland | Survey  6 Cantons in Switzerland covering 42 % of the adult pulmonary TB cases notified.  N = 162  Pulmonary TB only. | 46% initially consulted a general practitioner, 26% hospital.  Patient delay - No clear predictors  Longer health service delay - presence of fever (1.6 weeks, 95% confidence interval [CI] 0.5 to 2.6 weeks), having visited first a general practitioner or a paediatrician (1 week, 95% CI 0.1 to 1.9 weeks), having seen three or four doctors before beginning TB treatment (2.9 weeks, 95% CI 0.7 to 5.1 weeks).  Shorter health system delay were: having an X-ray at the first consultation (-2.9 weeks, 95% CI -4.8 to -0.9 weeks); and being male was marginally significant (-2.6 weeks, 95% CI -5.4 to 0.1 weeks). | Relevant (to PTB) and Robust. Representative sample of significant proportion of all Swiss PTB patients. |
| Bojovic, 2018  Montenegro | Retrospective cohort study  Patients (>15yrs) treated for TB in the specialized hospital for Lung Diseases in Brezovik, between Jan 1st 2015 and June 30th 2016.  N = 130  Pulmonary and extra-pulmonary TB. | Longer patient delay - being married (OR = 2.54, p = 0.026) having more negative attitudes towards tuberculosis (OR = 4.00, p = 0.045)  Shorter patient delay were greater knowledge of TB (some delay OR = 0.24, p = 0.031, extreme delay (OR = 0.30, p = 0.012)  Longer health system delay - a negative sputum smear some delay (OR = 7.01, p<0.001) or extreme delay (OR = 4.40, p = 0.032), being older than 47 years of age (OR = 2.61, p = 0.042), and specialist consultation delay (Some delay: OR = 1.08; extreme delay: OR = 1.05). |  |
| Chakma, 2022  Portugal | Retrospective cohort study  National surveillance data 2008-17  Healthcare delay: N = 11,430  Patient delay: N = 10,425  Pulmonary and extrapulmonary TB | Longer patient delay -15-64 year olds  Longer diagnostic delay – females |  |
| Dale, 2017  Australia | Retrospective cohort study  Victoria, Australia.  N=4867  Diagnosed with active TB pulmonary and extrapulmonary | Patient delays - similar male/female for pulmonary TB, some evidence that males with extra-pulmonary TB attended health care sooner after symptom onset than females (median delay of 15 days in males vesus 23 days in females).  Health system delay – for those with pulmonary TB 22 days in males versus 26 days in females.  Extra-pulmonary TB no difference health system and diagnostic delays between genders. | Authors note that males exhibit more severe TB disease, and so start treatment more promptly.  Differences reported were generally small-to-moderate and may have limited importance in a clinical setting. |
| Ekinci, 2014  Turkey | Cross-sectional survey  Referral hospital patients in Turkey >15yrs of age.  N = 136  Pulmonary TB, divided into smear negative and smear positive groups | Smear-negative and smear-positive tuberculosis patients similar rate of patient delay.  Longer healthcare service delay associated with being smear negative. | Relevant for patient delay but findings on health system delays may not be generalisable as this was a single centre, specialist chest/TB hospital in capital city |
| El Halabi, 2021  USA | Retrospective cohort study  Patients taken from a national private health-care claims database.  N = 738  Active TB, including pulmonary and extrapulmonary. | Longer healthcare system delays (first visit to initiation of treatment) - older age (8.4% per 10 year increase [95% CI 4.0 to 13.1]; p<0.0086) and non-HIV immunosuppression (19.2% [15.1 to 60.0]; p=0.0432).  Shorter delays - presenting with three or more symptoms (-22.5% [-39.1 to -2.0]; p=0.0415), use of chest imaging (-24.9% [-37.9 to -8.9]; p<0.0098), tuberculosis nucleic acid amplification tests (-19.2% [-32.7 to -3.1]; p=0.0241), care by a tuberculosis specialist provider (-17.2% [-33.1 to -22.3]; p<0.0087). | Robust study but relevance may be reduced as this was patients in private insurance healthcare system for minimum 8 months only. Likely bias away from experience of typical TB patient who is unlikely to have insurance. |
| Evenden, 2019  UK | Retrospective cohort study  London surveillance data between 2012-2018, adults ≥18yrs  N = 7216  Pulmonary TB adults | Patient delay (termed presentation delay symptoms to first visit) -  female (adjusted OR (aOR)=1.21; 95% CI 1.04 to 1.39), increasing age (aOR=1.004; 95% CI 1.001 to 1.008), white compared to Asian ethnicity (aOR=1.35; 95% CI 1.12 to 1.62), previous imprisonment (aOR=1.66; 95% CI 1.22 to 2.26), alcohol misuse (aOR=1.44; 95% CI 1.04 to 1.89).  Health service delay (visit to treatment) - female (aOR=1.39; 95% CI 1.21 to 1.59), increasing age (aOR=1.014; 95% CI 1.009 to 1.018), white ethnicity (aOR=1.41; 95% CI 1.19 to 1.68). | Included all London UK adult cases 2012-2018. TB incidence in London greater than rest of UK |
| Han, 2024, USA | Retrospective cohort study  Patients diagnosed with TB and Covid-19 in <120 days in California.  N = 58.  Pulmonary and extrapulmonary TB. | Total delay – median 29 days (IQR: 5-95). Total delay >30days was more common in patients with indicators of more severe TB (AFB smear positive, cavitary imaging results, disseminated pulmonary disease), or who were diagnosed with Covid-19 during a period of high local Covid incidence. | Multi-institution cohort study in similar incidence country but specific population subgroup (recent Covid-19 infection) |
| Jurcev-Savicevic, 2012/2013  Croatia | Cross-sectional study  7 Croatian counties between April and December 2006, including consecutively diagnosed adults (15 years or older).  N = 241  Pulmonary TB. | Reduced patient delay - symptoms (cough, losing weight), higher education.  Increased health service delay – female, smear-negative |  |
| Kato, 2012  Japan | Survey  Adult TB patients from 17 health centres in Japan.  N = 53  Pulmonary TB patients only. | Patient delay - presence of sputum and hemoptysis, positive sputum smear, low priority given to health, lack of a family physician, lack of consultation, taking over-the-counter drugs, and disliking hospital visits. | Small n and excluded asymptomatic and non-Japanese, only PTB. |
| Loutet, 2018  UK | Retrospective cohort  National TB surveillance data 2012-15  N = 7612  Pulmonary and extrapulmonary TB. | Total delay (symptom onset to treatment) - being female, aged 45 years and older, residing outside of London and having extra-pulmonary TB disease (aOR = 1.2, 1.2, 1.2, 1.3, 1.8, respectively) |  |
| Miller, 2021  USA | Retrospective cohort  National health insurance provider database  N = 3371  Patients diagnosed with primary, pulmonary, respiratory or miliary TB. Excluded extrapulmonary. | A considerable proportion of patients experienced multiple visits representing missed opportunities to diagnose TB - 23.8% of patients had more than five possible missed opportunities.  Diagnostic delay (Missed opportunities for diagnosis) – greater among individuals age ≥65 (OR of 1.262 CI 1.156 to 1.377), patients with a history of asthma (OR 1.331 (CI 1.138 to 1.557) or COPD (1.372 (CI 1.230 to 1.531), patients who had received chest imaging in the year prior to diagnosis (OR of 1.149 (CI 1.081 to 1.296) for chest CT and 1.231 (CI 1.121 to 1.353) for chest X- ray), patients who received fluoroquinolone in the year prior (OR 1.578 (CI 1.435 to 1.734)).  Misses were more likely to occur during weekend visits (1.495 (CI 1.272 to 1.758), in outpatient settings during periods of high influenza activity (1.259 (CI 1.052 to 1.507)), in an ED compared to outpatient setting (2.340 (CI 1.540 to 3.555).  Misses less likely to occur among patients in metropolitan locations (0.874 (CI 0.771 to 0.990), on only an inpatient visit (0.123 (CI 0.106 to 0.142), both an inpatient and outpatient visit (0.124 (CI 0.105 to 0.145), both an inpatient and ED visit (0.142 (CI 0.110 to 0.184)), or all three setting types (0.128 (CI 0.089 to 0.185)). | Relevance reduced as only commercially insured private health insurance population, registered at least 1 year pre diagnosis |
| Mor, 2013  Israel | Retrospective cohort study  Random sample of non-insured non-national migrants and insured Israeli citizens who were treated in the Tel Aviv TB outpatient clinic for active TB between 1998-2008.  N = 487  Pulmonary and extrapulmonary TB. | Patient delay (symptoms to medical consultation) longer for those who were a migrant, asymptomatic a smoker, had a co-morbidity.  Health service delay longer for those who were asymptomatic, had comorbidities. | Random sample from a single centre over 10 years and low relevance, old data (pre 2008) specific to Israeli versus migrant comparison |
| Morais, 2023, Portugal | Cross-sectional survey  TB patients >18yrs in Lisbon and Oporto (2019-2021)  N = 75  Pulmonary and extrapulmonary TB. | Patient delay – median 21 days (IQR: 11.5-63.5). Predictors of longer patient delay (≥21 days): no symptom valorization (adjusted Prevalence Ratio (PR) 1.59 (95% CI: 1.05, 2.42)). Patients who were more likely to disregard their symptoms were: smokers (PR 2.35, 95% CI: 1.14, 4.82). Patients less likely to disregard their symptoms were: richer (household income >1000 euros) (PR 0.39, 95% CI: 0.16, 0.94). | Survey based study in multiple areas. |
| Nunes, 2016  Portugal | Retrospective cohort  TB patients in Lisbon and Oporto, recorded by National TB Control Programme 2010-13  N = 2706 (Lisbon) and 1883 (Oporto)  Pulmonary and extrapulmonary TB. | Diagnostic delay (symptom onset (self-reported) to diagnosis)  Median 62 days (Lisbon) and 60 days (Oporto). Male sex and being HIV-positive predicted shorter diagnostic delay in both cities. Migrants had longer diagnostic delay in Lisbon only  Spatial variation  There was considerable variation between low-level administrative areas, suggesting that the cities should not be considered homogeneous | Possible recall bias, extreme delays excluded |
| Peri, 2018  Italy | Cross-sectional  Consecutive adult (>18yrs) patients diagnosed with TB between June 1^st^ 2011 and May 30^th^ 2012, at seven referral centres in Italy.  N = 137  Pulmonary and extrapulmonary TB. | Patient-related delay (symptoms to presentation)  Longer delays - those living shorter time in Italy (Odds Ratio [OR] 3.47; 95% Confidence Interval [CI] 1.09-11.01), symptoms underestimation marginally associated with longer delay (adjusted OR 3.43; 95% CI 0.98–12.04; p = 0.055), older age possible additional factor (median difference 0.05; 95% CI 0.03–0.09; p = 0.019).  Patient-reported reasons for delay - mild nature of symptoms (82%), good self-perceived health (76%). A significant, proportion of patients (17 to 32%), reported fear of the consequences of a diagnosis, fear of being reported to the authorities, lack of knowledge of the health system, time and economic constraints. Wrong beliefs and poor knowledge of TB not significantly associated with longer delay. Around two thirds had good knowledge about the disease, 21% reported self-blame, 27% blamed others, 26% believed people with TB should be excluded from society. Extrapulmonary not significantly different to pulmonary in regard to patient delay. No significant association between patient-related delay and age, gender, school degree, occupation, annual income, housing conditions or nationality.  Diagnostic delay (over 10 weeks) - Extra-pulmonary TB (OR 4.3; 95% CI 1.4-13.8), absence of respiratory symptoms (OR 2.57; 95% CI 1.19–5.55; p = 0.016), first contact with general practitioner (OR 5.1; 95% CI 1.8-14.5) longer delay. Healthcare delay not associated with other covariates including patient nationality, demographic and social characteristics. Those who sought medical care from their general practitioner consulted an average of 2.8 healthcare providers before diagnosis, hospital-based Emergency Department mean of 1.8 consultations before diagnosis, first evaluated by a specialist consultant in outpatient clinics diagnosed after mean 1.4 consultations. | Patient self-reported questionnaire |
| Pezzotti, 2015  Italy | Survey  Adult patients (≥ 18 years) diagnosed between September 2010 – September 2011 in the Lazio region.  N = 278  Pulmonary TB only. | Median patient delay (PD; symptom onset to consultation), health service delay (HSD; consultation to diagnosis) and total delay (TD) were 31, 15 and 77.5 days, respectively.  All types of delay were shorter for patients born outside Italy. Factors associated with longer delay were absence of fever and presence of weight loss for PD; prior unspecific treatment, absence of cough, consultation with a general practitioner, visit at an outpatient clinic and a PD <30 days for HSD. | Relatively small sample and old data |
| Quattrocchi, 2018  Italy | Cross sectional survey  Cases diagnosed at 30 centres in four regions, 2014-16; 55.7% born outside Italy  N = 253  Pulmonary TB only. | Median PD, HSD and TD were 30, 11 and 45 days, respectively.  Patient delay  Factors associated with longer patient delay were stigma, chest pain, weight loss, paying for transportation and distance to the health centre (the latter three were also associated with TD).  Health system delay and Total delay  Shorter health system delay was associated with foreign-born and female status, dizziness and seeking care at a hospital. Prior unspecific treatment was associated with longer HSD and TD. Haemoptysis and repeated visits with the same provider predicted shorter TD. | 255/344 patients participated; possible recall bias and selection bias, especially for ‘foreign-born’ patients |
| Roberts, 2020  UK | Retrospective cohort  All cases 2011-2015 resident in South East England.  N = circa. 600-900 for different aspects of the analysis.  Pulmonary TB only. | Longer patient delay associated with language barriers (40% longer presentation delay time ratio 1.40, 1.01–1.94) and mental health barriers (time ratio 2.06 (1.22-3.5)). Shorter patient delay associated with age group 0-14yrs (TR 0.23, 0.09-0.59).  Shorter healthcare delay was associated with positive sputum smear (time ratio 0.58, 0.47–0.70), UK residency <2 years (TR 0.47, 0.32–0.67), male sex (TR 0.74, 0.60–0.91), secondary care referral (TR 0.63, 0.51–0.78), and presence of a social risk factor (TR 0.59, 0.38-0.94). Most healthcare delay was related to time to refer to specialists. |  |
| Saldana, 2013  UK | Retrospective cohort  Patients with TB resident in Thames Valley who started treatment in 2007  N = 273  Pulmonary and extrapulmonary TB. | The median time between symptom onset and initiation of treatment was 73 days [95% confidence interval (CI) 65-89], of which the contributions of health service, patient and referral delays were 39 (95% CI 34-55), 29 (95% CI 22-36) and 16 (95% CI 12-24) days, respectively.  Patient delay  On univariate analysis, only male gender was significantly associated with longer patient delay  Health system delay  On multivariate analysis, extrapulmonary TB, female and UK-born patients were associated with longer health service delay.  Total delay  Age and extrapulmonary TB (P ¼ 0.010) were associated with longer overall delay. | UK study but old data and relatively small sample |
| Salinas, 2012  Mexico | Cross-sectional  New diagnoses in Coahuila, between 2008-2009.  N = 458  Pulmonary TB only. | Patient factors – Longer delays associated with lack of formal education (P = 0.050) and living more than 5 km from a health unit (P = 0.034).  Health system factors - longer when the first consultation was with a private physician (P < 0.001) and when patient age was >=46 years (P = 0.001). | Relevance to UK affected by country income (middle) and TB burden (>3x that of UK @24/100,000) |
| Santos, 2021 a/b  Portugal | Retrospective cohort study  National surveillance data 2008-2017  N = 11,762 pulmonary TB  Pulmonary TB only. | Greater patient delays - being from a high TB incidence country. alcohol abuse, unemployment, patients with respiratory disease, oncologic diseases.  Greater diagnostic delays - females, people of older age (over 45), high TB incidence country.  Both patient delay and healthcare delay increased over time in lower incidence areas. |  |
| Soares, 2022  Portugal | Ecological study  All cases from national surveillance system 1^st^ January 2008 – 31^st^ December 2017, including 278 municipalities  N = 278 municipalities (ecological study)  Pulmonary TB only. | Higher delay areas (mean 108 days between symptoms and diagnosis) - smaller population density, smaller proportion of unemployed, fewer health centres and higher old-age dependency ratio, and greater proportion of men, TB incidence, immigrants and high school dropout. | Less relevant to UK than main study (Santos et al. 2021) as this compares delay-related factors in regions of Portugal that may be driven by country specific issues. |
| Stjepanovic, 2018  Serbia | Retrospective cohort  Adult (≥ 18 years) patients hospitalised with TB between March 2015 to December 2015 at the Department of Pulmonology, Clinical Center Serbia.  N = 100 patients hospitalised with TB  Pulmonary and extrapulmonary TB. | Patient delay – Patient delay accounted for a substantial part (78.4%) of total delay of 118 days.  Longer delay associated with excessive alcohol consumption (r=0.362, p <0.001), number of cigarettes (r=0.314, p=0.001). Patients with negative family history of TB more likely to delay seeking help.  No significant effect on delay - the presence of symptoms (p>0.05), TB category and chest radiograph abnormalities (p>0.05). | Small single hospital centre study |
| Tattevin, 2012  France | Cross-sectional study  Patients notified to health authorities between April to June 2010.  N = 225  Pulmonary and extrapulmonary TB. | Reduced diagnostic delay - medical insurance (OR 0.24, P = 0.014) and previous TB (OR 0.28, P = 0.049).  Patient delay – Less patient delay being followed by a GP (OR 0.25, P = 0.006). |  |
| Türkkani, 2020  Turkey | Nationwide survey  Smear-positive pulmonary TB in patients diagnosed between January 1^st^ and December 31^st^ 2018.  N = 853  Pulmonary TB only. | Patient delay - no association between sex, age, literacy, residential location, the presence of chronic respiratory diseases, and patient delay. Patient delay was shorter for patients with hemoptysis, fever, dyspnoea, and chest pain. Longer for smokers. |  |
| Vigneswaran, 2023, Australia | Retrospective cohort study  Adult inpatients diagnosed with TB at Royal Darwin hospital from 2010 to 2020.  N = 84  Pulmonary and extrapulmonary TB. | Patient delay – median 53 days.  Predictors of longer patient delay: extra-pulmonary TB (median delay: 100 days (IQR: 90-105)) compared with pulmonary TB (median delay: 39 days (IQR: 27-54), and TB smear negative (median delay: 69 days (IQR: 57.5-90) compared with various stages of smear positive.  Health service delay – median 21 days.  Predictors of longer healthcare delay: aged ≥45yrs (median delay: 30 days (IQR: 16-51) compared to aged <45yrs (median delay: 14 days (IQR: 8-30).  Total delay – median 90 days.  Predictors of longer total delay: extra-pulmonary TB and TB smear negative.  Only extrapulmonary disease remained as a statistically significant predictor in the multivariable analysis. | Single institution cohort study in a similar incidence country but in unique setting within Australia with significant numbers of First Nation’s peoples and migrants. |
| Wang, 2011  Canada | Retrospective cohort  Active TB on the British Columbia Linked Health Database between 1997 and 2006.  N = 1544  Pulmonary TB only.  Patients with active TB, national database | Sex, age, foreign-born status and socio-economic status were non-associated with health service delay. Health service delay increased with the number of antibiotic courses received (not the type of antibiotic). Delay increased by factor of 2.10 for patients prescribed antibiotics.  Health service delay reduced if HIV positive. | Old data 1997-2006 |
| Williams, 2018  Australia | Retrospective cohort  Adult (≥ 18 years) patients commenced on treatment between 1^st^ December 2011 – 1^st^ December 2014 at a tertiary teaching hospital in Western Melbourne.  N = 133  Pulmonary TB only. | Delays between symptom onset and treatment commencement are predominantly due to patient delay in presentation for care. Patient delay median of 28 days from symptom onset until first health service contact, median health system delay was 18 days.  Patient delay (symptoms to healthcare contact more than 35 days) – greater delay associated with being from a country with an annual TB incidence of <50/100 000 (OR 5.98, 95% CI 1.19, 29.98), diabetes mellitus (OR 3.02, 95% CI 1.04, 8.78, weight loss (OR 2.23, 95% CI 1.00, 4.96) 95% CI 1.04, 8.78).  Less delay - being Australian-born or resident in Australia ≥6 years (OR 0.03, 95% CI 0.12, 0.74; OR 0.30, 95% CI 0.00, 0.033.  Health system delay (more than 21 days) greater delay associated with patient age 65 years or older (OR 3.07, 95% CI 1.21, 7.79) patient review in outpatient clinic (OR 7.67, 95% CI 3.51, 16.77).  Lesser delay associated with presence of cough (OR 0.09, 95% CI 0.01, 0.72), review in the emergency department (OR 0.33, 95% CI 0.16, 0.69) or admission to hospital (OR 0.17, p 95% CI 0.06, 0.46),  AFB smear positivity (OR 0.23, 95% CI 0.09, 0.56), NAT positivity (OR 0.14, 95% CI 0.03, 0.62), cavitating lesions on CXR (OR 0.25, 95% CI 0.09, 0.67). | Single tertiary centre study |
| Zão, 2019  Portugal | Retrospective cohort.  Cases notified between 2010 and 2014 in Portugal  N = 6838  Pulmonary and extrapulmonary TB. | Median patient and healthcare system delays were 33 and 17 days, respectively.  Patient delay  Adjusted regression models revealed that longer patient delay occurred in patients born outside Portugal and those addicted to alcohol and drugs.    Health service delay  Longer healthcare system delay was observed among patients with extra-pulmonary TB and pulmonary comorbidities (lung cancer, sarcoidosis or COPD) and in patients living further from a healthcare service. |  |

## Extraction Table 3: qualitative studies

| First author, date  Country of origin | Study design  Sample size  Population  Pulmonary (P) or non-pulmonary (NP) | Key themes (points in pathway, main obstacles) | Comments re relevance /robustness |
| --- | --- | --- | --- |
| Aguiar, 2024, Portugal | Semi-structured interviews, N = 21. Medical doctors treating TB patients in prisons. | Promoters of effective diagnosis and treatment  Doctor’s reported good flow of clinical information between institutions as a contributing factor to effective diagnosis and treatment.  Barrier to diagnosis  Low level of health literacy among inmates. | Majority of study is focused on impact of specific TB protocol in Portugal. |
| Craig, 2014  UK | Interview study  N = 17  Patients attending a major TB centre in London, mostly homeless and with complex health and care needs | Barriers to seeking care  Participants demonstrated some knowledge of tuberculosis but their awareness of personal risk was low. Symptoms commonly associated with tuberculosis were either not recognised or were attributed to other causes. Many accessed health care by chance and in some cases for health concerns other than tuberculosis.  Most participants were drug dependent and concern about forced abstinence was a further obstacle to seeking care. | Small sample of patients with particularly complex needs |
| Gerrish, 2013  UK | Interviews  N=14 + 18  TB patients from Somali who had received treatment and practitioners | Patient delays – patients perceived that they presented soon after onset of symptoms, some experienced symptoms associated with pulmonary TB, others had more diffuse symptoms. Felt that their concerns had not been treated seriously, some perceived slow referral when symptoms persisted.  Healthcare system delays - low clinical suspicion of tuberculosis among general practitioners. Most patients reported frustration at the time it took from first visiting their GP to a diagnosis of TB being confirmed. They reported visiting their GP repeatedly and being prescribed analgesia and/or antibiotics. Practitioners acknowledged that diagnosing TB could be challenging when patients presented with atypical symptoms, harder if in a practice where few cases seen each year. Language barriers could make taking a history challenging. Practitioners perceived some patients presented late due to stigma in the community, perception that GPs were becoming more aware. |  |
| Ribero, 2021  Portugal | Interviews  N=27  TB patients attending a treatment centre  Average age 38, 20 recent migrants, 11 portugese nationality, more extra- pulmonary | Healthcare seeking behaviour model (health belief model) – delay classified as patient delay over one month, health system delay over two months.  Patient delay – symptoms unspecific, perceived benefits of seeking healthcare low, and low perceived severity of disease, until a strong event (cue to action) induced them to act. Those seeking help had awareness of symptoms and, accessibility, and trust in different entry points of the healthcare system. Low perceived barriers to seeking healthcare combined with a high perceived severity of disease results in timely healthcare seeking behaviour. Preference for entry points based upon satisfaction, familiarity, accessibility, informal knowledge, literacy, economic capacity. Cultural factors such as being recognised or stigma may have contributed to ER preference. Nationals and migrants showed similar behaviour patterns.  Healthcare system delay - longer for participants opting for private care, typical diagnosis in ER after prolonged deterioration, unspecific TB symptoms. Delay longer for extra pulmonary TB, delays longer for those first visit to a GP. A TB screening programme, an occupational health service, a specialist consultation, an ambulance service, and a charitable organisation assisted participants to getting a diagnosis. Most participants did not have an assigned primary care practitioner which may have led to access via an ER. |  |
| Sagbakken, 2010  Norway | Interviews  N= 22  Immigrants from Somalia or Ethiopia, diagnosed with TB and treatment initiated, half male/female | Patient-related – longer delay associated with persistent cough not accompanied by symptoms such as: weight loss and weakness; mild, diffuse, atypical, and/or intermittent symptoms; and a sense of not being a likely victim of TB. Few knew TB could be anything but related to lungs, perception of a skinny weak person coughing, caused by poverty and poor diet (therefore not perceiving self as vulnerable to TB). Some described suspecting TB due to fatigue, losing weight, night sweats having seen these symptoms in others.  Health system – shorter delays associated with a medical history that gave suspicion of TB, presenting with typical TB symptoms, or being screened for TB at arrival. Diagnosis could be difficult to confirm with health professionals not associating difficulties with symptoms of TB. This led to delays in initiating diagnostic tests for TB. Delay in testing was particularly for cases of extra-pulmonary TB. Few participants were referred for a chest x ray or sputum test on first presentation. Those with extra-pulmonary described declining health for some time, presented with diffuse and non-specific symptoms, lack of awareness of typical and atypical symptoms amongst health professionals. |  |
